# Dynamic deuterium metabolic imaging in glioblastoma at 7T

**DOI:** 10.1007/s10334-025-01299-3

**Published:** 2025-10-09

**Authors:** Narjes Ahmadian, Mark Gosselink, Sigrid Otto, Dimitri Welting, Kiki Tesselaar, Tom Snijders, Pieter van Eijsden, Jeanine Prompers, Dennis Klomp, Evita Wiegers

**Affiliations:** 1 Center for Image Sciences, University Medical Center Utrecht, Heidelberglaan 100, 3584 CX Utrecht, Netherlands; 2 Department of Neurology & Neurosurgery, Brain Center, University Medical Center Utrecht, Utrecht, Netherlands; 3 CTI Lab Support, University Medical Center Utrecht, Utrecht, Netherlands; 4 Human Biology, NUTRIM School of Nutrition and Translational Research in Metabolism, Maastricht University Medical Center+, Maastricht, Netherlands

**Keywords:** Glioblastoma, Deuterium Metabolic Imaging, Glutamate/Glutamine (Glx), Lactate

## Abstract

**Objective:**

This study investigates the dynamic metabolic characteristics of glioblastoma (GBM) using Deuterium Metabolic Imaging (DMI) at 7 T, aiming to dynamically characterize the Warburg effect in vivo.

**Material and methods:**

Five newly diagnosed GBM patients underwent dynamic DMI prior to any treatment. 3D ^2^H free-induction-decay (FID)-Magnetic resonance spectroscopy imaging (MRSI) measurements (11:44 min per scan) were performed at 7 T during ~ 100 min following [6,6’-^2^H_2_]glucose consumption. Venous plasma glucose and plasma ^2^H-Glc atom percent enrichment (APE) levels were measured during the scan. Brain ^2^H-glucose (^2^H-Glc),^2^H-Glutamate/Glutamine (^2^H-Glx), ^2^H-Lactate (^2^H-Lac), ^2^H-Lac/^2^H-Glx were analyzed with a two-level (time and tissue type) Linear Mixed Model.

**Results:**

Brain ^2^H-Glc levels were similar across tissue types. ^2^H-Glx was significantly lower in tumors compared to normal appearing brain tissue (NABT) (*p* < 0.01). ^2^H-Lac was significantly higher in tumors compared to NABT (*P* < 0.01). The ^2^H-Lac/^2^H-Glx ratio provided tumor-specific contrast, starting 40-50 min post [6,6’-^2^H_2_]glucose consumption. Venous plasma glucose and ^2^H-Glc APE increased within 50 min and venous ^2^H-Glc APE stabilized at ~ 60%.

**Discussion:**

Dynamic DMI at 7 T reveals metabolic alterations in GBM, particularly through the ^2^H-Lac/^2^H-Glx ratio. This contrast was primarily driven by decreased ^2^H-Glx rather than profoundly increased ^2^H-Lac. These findings support the utility of DMI in assessing metabolic reprogramming in brain tumors.

**Supplementary Information:**

The online version contains supplementary material available at 10.1007/s10334-025-01299-3.

## Introduction

Glioblastoma (GBM) is the most common primary malignant brain neoplasm in adults. The prognosis for GBM is generally poor [1], and despite research on new therapies, the survival rate has not changed significantly in recent times [2]. Better understanding of GBM pathophysiology is of utmost importance.

Metabolic reprogramming is one of the hallmarks of cancer [3–5]. In GBM, this typically involves a shift from oxidative phosphorylation to aerobic glycolysis, a process known as the Warburg effect [6, 7]. Metabolic reprogramming in brain tumors is a complex and multifactorial process [8, 9]. Imaging tumor metabolism may give insights into brain tumor pathophysiology [10]. Conventional MRI provides valuable structural and anatomical information about lesions and is used to monitor patients before, during and after treatment. However, the nature of a tumor may not always be captured when focusing on gross structural patterns [9, 11]. Due to the known uncertainties regarding the accuracy of conventional MRI for noninvasive assessment of brain lesions, various efforts have been made to enhance brain tumor diagnostics through metabolic imaging using PET (positron Emission Tomography) and Single-photon emission computed tomography (SPECT) [12–14]. Fluorodeoxyglucose (^18^FDG)-PET can image glucose demand of a tumor, but provides no information regarding the metabolic pathways through which glucose is used [15].

The recently developed ^18^F-labeled amino acid O-(2-^18^F-fluoroethyl)-l-tyrosine (^18^F-FET)-PET has gained attention for its ability to image the accumulation of the radiolabeled amino acid tracer in tumor cells [15]. ^18^F-FET-PET shows lower uptake in normal appearing brain tissue than FDG-PET, resulting in less background signal and providing a clearer contrast between tumor and healthy brain compared to FDG-PET [16]. ^18^F-FET-PET has been shown to be particularly useful in distinguishing tumor recurrence from radiation-induced changes in cases of suspected recurrent high- and low-grade gliomas [10, 16]. However, it still involves the use of a radioactive isotope and its diagnostic value is still imperfect; of note, ^18^F-FET-PET does not assess metabolic pathways such as oxidative phosphorylation, and therefore does not capture the Warburg effect.

Deuterium metabolic imaging (DMI) is a novel MRI technique. Deuterium (^2^H) is a non-radioactive isotope of hydrogen with a low natural abundance of ~ 0.01% [17, 18]. Oral consumption of deuterated glucose leads to ^2^H-labeling of glucose (^2^H-Glc), lactate (^2^H-Lac) and glutamate and glutamine (^2^H-Glx) in the brain, which can be detected with DMI [17–19]. Using DMI, it was shown that the ratio of ^2^H-Lac over.^2^H-Glx results in a tumor-specific contrast, which may represent the Warburg effect [17, 21–23]. Furthermore, a pre-clinical study by Low et al. revealed that DMI could be used to differentiate between metabolic subtypes of GBM and to monitor responses to chemoradiation within 24 h [24]. The ability of the non-invasive and non-ionizing DMI to provide a detailed metabolic map makes it a promising tool for future cancer research and clinical applications [17, 21]

While the time courses of label incorporation into ^2^H-Glc, ^2^H-Glx and ^2^H-Lac have been studied in preclinical GBM models after deuterated glucose infusion using surface coil localization[24], human DMI studies in GBM patients have so far been limited to spatially resolved but single timepoint measurements, typically ~ 45 min after oral consumption of deuterated glucose. Dynamic DMI allows continuous monitoring of metabolic changes over time, capturing real-time glucose uptake, its conversion into lactate, and oxidative metabolism. This approach may offer a more comprehensive understanding of tumor metabolism, aiding in differentiating tumor from normal tissue and providing valuable insights into metabolic heterogeneity between tissue types. Here, we aim to detect and dynamically characterize the Warburg effect in GBM patients using dynamic DMI at 7T.

## Material and methods

### Experimental protocol

The study protocol was approved by the medical ethical committee NedMec (NL83501.041.23), Utrecht, the Netherlands. The study was performed in accordance with the ethical standards as laid down in the 1964 Declaration of Helsinki and its later amendments. Informed consent was obtained from all patients.

Five newly diagnosed GBM patients were included pre-operatively, based on the preoperative suspicion of a GBM, which was later confirmed histologically. Exclusion criteria were [1] contraindications for 7 T MRI [2] pregnancy or lactating [3] the presence of diabetes mellitus type 1 or 2 using antidiabetic medication and [4] Karnofsky Performance Scale (KPS)(i.e., a standardized tool used to measure a patient’s functional status and ability to perform daily activities, ranging from 100 (normal functioning) to 0 (death)) score below 70. Participants were scanned before the start of any treatment, except for the use of dexamethasone, which was started per clinical protocol 2–7 days prior to the scan.

Prior to the scan, [6,6’-^2^H₂]glucose was dissolved in water using a magnetic stirrer for up to 20 min. During this time, the solution was intermittently stirred and visually monitored until it became fully clear and colorless.

All patients fasted at least 6 h prior to the scan; four fasted overnight, while one had a light breakfast before 07:00 AM and was scanned at 02:00 PM. They received an intravenous (IV) catheter for continuous blood sampling every 10 min.

### DMI hardware and acquisition

DMI data was acquired with a 7 T MR scanner (Philips, Best, NL), using a custom-built dual tuned ^31^P/ ^2^H transmit bore coil (with a diameter of 60 cm and a length of 40 cm, integrated behind the bore of the MRI) [25] and a head coil equipped with eight ^2^H trapezoid shaped receive loop coils (7 loops of 200 × 75–120 mm and 1 smaller frontal loop of 120 × 75–180 mm, and 6 mm wide copper traces) which were placed around a helmet, combined with eight ^1^H transmit/receive dipole antennas [26] positioned at a minimal distance of 35 mm from the ^2^H receive coils.

Prior to [6,6’-^2^H_2_]glucose administration, B0 shimming was performed, a T_1_w image (voxel size: 0.5 × 0.5 × 1.0 mm^3^, field of view (FOV) = 251 (Anterior–Posterior (AP)) × 180 (Right-Left (RL)) × 200 (Feet-Head (FH)) mm^3^, echo time (TE) = 1.97 ms, repetition time (TR) = 8 ms, flip angle (FA) = 6°, and number of averages (NAV) = 2) and a baseline DMI scan were acquired. This was followed by the oral consumption of 0.50 g/kg body weight [6,6’-^2^H_2_]glucose through a 1.5 m tube, while subjects remained in the MR system. DMI data acquisition lasted ~ 75–100 min after [6,6’-^2^H_2_]glucose ingestion (depending on the subject’s compliance).

DMI data was acquired using a 3D free induction decay (FID)- Magnetic resonance spectroscopy imaging (MRSI) sequence with a 40˚ block pulse (B_1_^+^  = 10 μT) and Hamming weighted k-space sampling. Acquisition parameters were as follows: nominal voxel size:12 × 12x12 mm^3^, FOV: 240 × 180x216 mm^3^, matrix size is 20 × 15 × 18, TR: 100 ms, TE: 1.82 ms, spectral bandwidth: 2800 Hz, 256 data points, number of averages at the center of k-space: 4 (gradually reduced to 1 number of averages in the outer regions of k-space), and an acquisition time of 11:44 min/scan.

### Data processing

In-house developed MATLAB scripts (Matlab R2021a, MathWorks, USA) were used for data processing, which included spatial Fourier transformation and phase correction. We also applied a correction for the Hamming-weighted acquisition pattern: the acquisition used 4 averages in central k-space and 1 in outer regions, with a standard Hamming function applied along all phase-encoding directions. Continuous coefficients were scaled to [1, 4], rounded to integers, and later corrected to match the ideal Hamming profile. Data from the 8 ^2^H receive channels was combined with Whitened singular-value decomposition (WSVD) [27]. After coil-combination, principal component analysis-based (PCA)-denoising per timepoint with a patch size of 5 × 5x5 [28], apodization with a 5-Hz exponential function and spectral zero-filling to 2048 points was applied [29].

The spectra were fitted with Accurate, Robust and Efficient Spectral fitting (AMARES) using the OXford Spectroscopy Analysis (OXSA) toolbox in Matlab, using Lorentzian line shapes, in two steps [30]. First only the deuterated water (HDO) signal was fitted as a singlet with a variable frequency (4.45 to 4.95 ppm) and variable zero-order phase (0 to 360 degrees). There were no restrictions set for linewidth and amplitude (0 to infinity). The estimated frequency and zero-order phase were used to correct for frequency and zero-order phase shifts per voxel for all timepoints. Subsequently, the phase- and frequency-corrected data were used to fit all four metabolites signals (HDO, ^2^H-Glc, ^2^H-Glx and ^2^H-Lac). Any potential residual frequency offsets and/or zero-order phase errors were addressed by allowing a range of ± 0.1 ppm for the frequencies and ± 20 degrees for the zero-order phase, separately for each metabolite signal. Again, there were no restrictions set for linewidth and amplitude (0 to infinity).

Only voxels where HDO in the baseline DMI scan (i.e., before [6,6-^2^H_2_]-glucose consumption) was fitted with a Cramèr-Rao Lower Bound (CRLB) < 10% were taken into account for all consecutive datasets. For ^2^H-Glc, ^2^H-Glx and ^2^H-Lac a CRLB < 50% was used as a cut-off value at each time point. The fitted baseline amplitude (i.e., before [6,6’-^2^H_2_]glucose ingestion) of ^2^H-Lac was subtracted from each subsequent scan to correct for (extracranial) lipid contamination. The analysis was repeated without this lipid correction to assess its impact on the results.

The fitted metabolite signals were normalized to the baseline HDO signal amplitude per voxel and corrected for ^2^H-label loss [31] and differences in metabolite T_1_-relaxation times (i.e., ^2^H-Lac: 159 ms; ^2^H-Glx: 149 ms; ^2^H-Glc: 66 ms and HDO:320 ms) [32], as described previously [33].

In our quantification, we corrected for T_1_-relaxation times per metabolite using the following equation:

$${R}_{M}=\frac{1-cos\left(FA\right)*{e}^{-\frac{TR}{{T1}_{M}}}}{\mathrm{sin}\left(FA\right)*(1-{e}^{-\frac{TR}{{T1}_{M}}})}$$

where FA refers to the flip angle, TR to repitition time and the T_1M_ to T1 values per metabolite (M) reported in normal brain tissue at 7 T [32]. The correction factors (R_M_) are 1.97 ms, 1.93 ms, 1.65 ms and 2.54 ms for ^2^H-Lac, ^2^H-Glx, ^2^H-Glc and HDO, respectively, assuming.

The T_1_w image was segmented in gray matter (GM), white matter (WM), and cerebrospinal fluid (CSF) using Statistical Parametric Mapping (SPM)-12, to accommodate voxel-wise fractional water content correction (GM: 078, WM: 0.65, CSF: 0.97 [34]). The tumor and tumor core were manually delineated on the T_1_w images acquired at 7 T. The tumor core was defined as the central part of the tumor, i.e., the hypointense region visible on the T_1_w image. This region corresponded to the contrast-enhanced (CE) area observed on the standard clinical T_1_w image at 3 T following gadolinium injection, which was made earlier. The T_1_w CE image at 3 T is not directly used for delineation but served as a reference scan. We assumed a fractional water content of 0.85 for tumor tissue [35]. A voxel was assigned as “Tumor” or “TumorCore” if at least 50% of its volume overlapped with the mask. Normal appearing brain tissue (NABT) was defined as the total brain mask minus Tumor/TumorCore ROI. Mean ^2^H-Glc, ^2^H-Glx ^2^H-Lac and ^2^H-Lac/^2^H-Glx levels were calculated per timepoint per ROI.

Only for visualization purposes, the metabolic maps were upscaled per slice by a factor of five using bicubic interpolation, followed by Gaussian smoothing (σ = 1.2). This resulted in a final display matrix size of 100 × 75 × 18 voxels. Unprocessed (i.e. without spatial interpolation) metabolic maps are provided as supplementary material.

### Blood sampling and analysis

Venous blood samples of 5 ml were obtained every 10 min from a peripheral venous catheter, placed in the median cubital vein. The arm with the IV catheter was kept warm with a heating pad set to ~ 37 °C, and a continuous NaCl drip was used between samplings to prevent clotting. All blood samples were collected while the participants remained in the scanner, allowing uninterrupted scanning and blood collection.

1 ml venous blood was used to immediately determine plasma glucose levels using a YSI glucose analyzer (2500 series, YSI, USA). The remaining 4 ml of the blood samples were stored on ice and were centrifuged at 4000 RPM for 10 min after each scan session. Plasma was then stored at -80 °C for later analysis of venous plasma ^2^H-Glc atom percent excess [APE] of deuterium within two weeks. Deuterated glucose enrichment in plasma was quantified using gas chromatography-mass spectrometry (GC–MS, Shimadzu QP2020NX), as described by Ackermans and Macallan et al. [33, 36, 37]. Plasma was deproteinized with methanol, and the methanol fraction containing deuterated glucose was derivatized to glucose aldonitrile penta-acetate for GC–MS analysis. A standard curve of labeled deuterated glucose was used to determine APE, based on the (m + 2)/m ion ratio (m/z 328 and m/z 330). A linear regression equation was applied to calculate enrichment from the (m + 2)/m ratios of controls and samples [18, 35, 36].

### Statistical analysis

Mean venous plasma ^2^H-Glc APE and mean venous plasma glucose levels between 50 and 90 min (plateau) of [6,6’-^2^H_2_]glucose ingestion was calculated between all patients. The serial venous plasma glucose and venous plasma ^2^H-Glc APE were compared between patients over the entire scan duration using a General Linear Model with Repeated measures.

Statistical significance of time and tissue (NABT, tumor and tumor-core) effects, and their interaction, on brain ^2^H-Glc, brain ^2^H-Glx, ^2^H-Lac and ^2^H-Lac/Glx levels were analyzed using a two level Linear Mixed Model, with time and tissue type as fixed factors. If the results of the tests were significant, Bonferroni Post-Hoc tests were conducted.

Statistical significance was set at *p* < 0.05. All statistical analysis was performed in IBM SPSS Statistics (version 27).

## Results

**Fig. 1:**
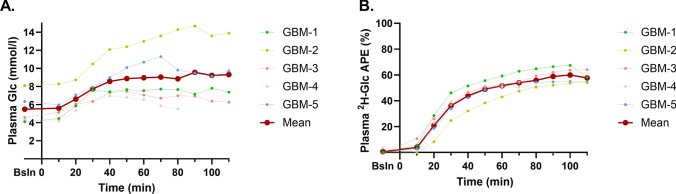
**A** Plasma glucose concentration and **B** Plasma ^2^H-Glc APE after oral consumption [at T = 0 min] of [6,6’-^2^H_2_]glucose in all patients. Each patient is represented by a dashed line, with the mean value shown in red. Bsln: baseline measurement

Five GBM patients (Table 1) underwent dynamic DMI prior to any treatment. Following [6,6’-^2^H_2_]glucose administration, venous plasma glucose levels and venous plasma ^2^H-Glc APE initially rose during ~ 50 min (Fig. 1). Venous plasma ^2^H-Glc APE stabilized at approximately 50–60% during the 50–90 min after [6,6’-^2^H_2_]glucose ingestion (Table 2). The mean venous plasma glucose level within this timeframe is the highest in patient 2, probably due to the highest dosage of dexamethasone (Table 2). There was a significant main effect of time on both venous blood plasma glucose levels and venous plasma ^2^H-Glc APE over the entire scan duration (both *p* < 0.01). However, there was no significant difference across subjects in the main time effect on venous plasma glucose and venous plasma ^2^H-Glc APE levels post [6,6’-^2^H_2_]glucose consumption (*p* = 0.91 and *p* = 0.72, respectively).

**Table 1:** Patients characteristics

	Sex	Age (years)		BMI (kg/m^2)^	Fasting plasma Glucose level (mM/L)	Tumor location	Dexamethason (mg/day)	Tumor type
GBM-1	M		44	22.4	4.11	L. temporal lobe	8	GBM, IDH wildtype
GBM-2	M		69	31.5	8.11	R. temporal lobe	12	GBM, IDH wildtype
GBM-3	M		73	22.7	4.58	L. frontal lobe	6	GBM, IDH wildtype
GBM-4	M		78	29.4	4.35	R. temporal lobe	4	GBM, IDH wildtype
GBM-5	M		63	28.9	6.34	R. frontal lobe	4	GBM, IDH wildtype

*M* male, *L* left, *R* right, *IDH* isocitrate dehydrogenase

**Table 2:** Mean and standard deviation [SD] of plasma Glc and plasma ^2^H-Glc APE within 60–100 min following [6,6’-^2^H_2_]glucose ingestion

	Plasma Glc Mean [SD] (mM)	Plasma ^2^H-Glc APE Mean [SD] [%]	
GBM-1	7.5 [0.2]		61.8 [4.4]
GBM-2	13.6 [0.9]		46.3 [5.7]
GBM-3	7.0 [0.3]		55.8 [4.8]
GBM-4	6.2 [0.6]		50.8 [0.5]
GBM-5	10.3 [0.7]		52.9 [2.6]

**Fig. 2:**
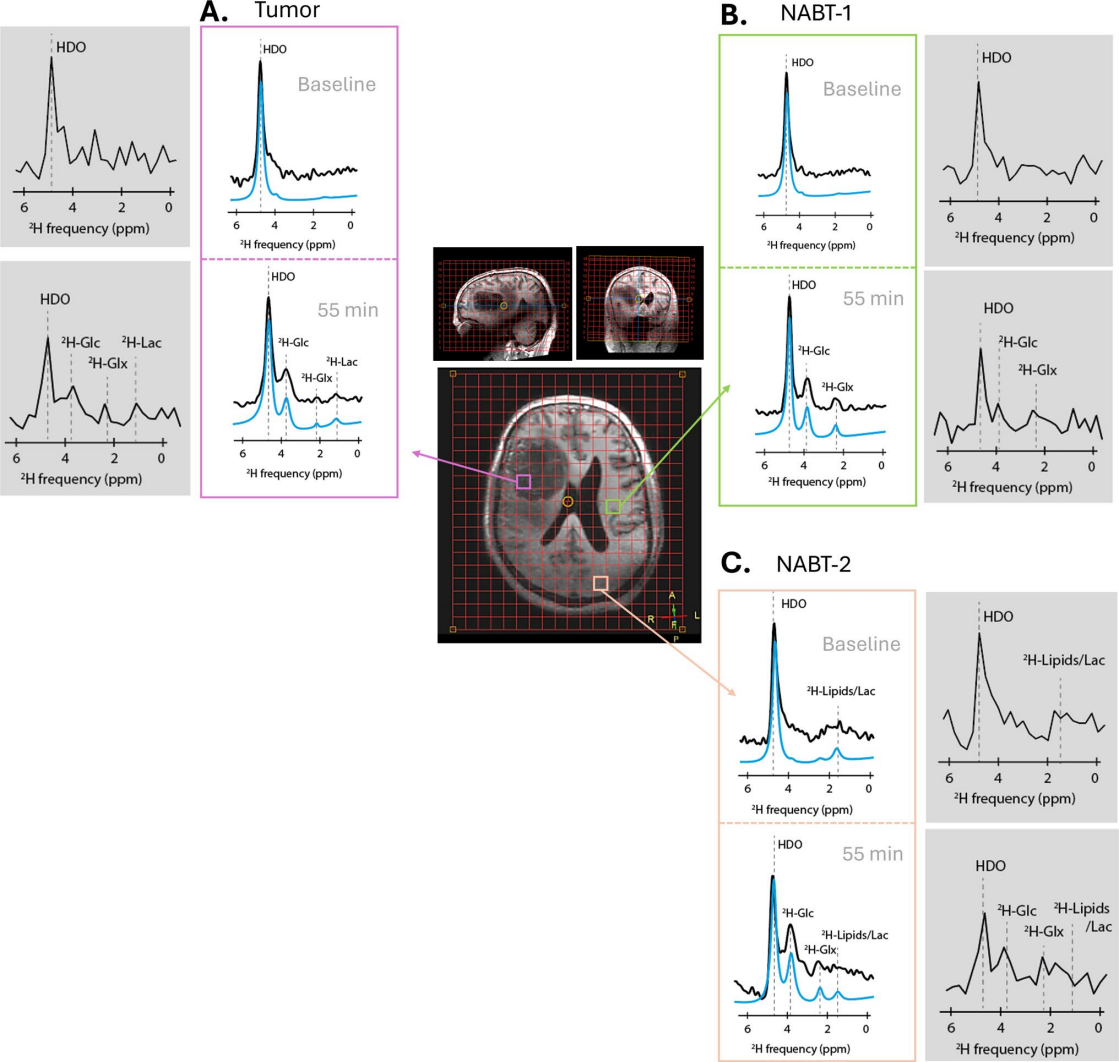
Examples of a T_1_w image of patient GBM-5 with the ^2^H-MRSI grid; ^2^H MR spectra in Tumor tissue (purple box) and two voxel within normal appearing brain tissue (NABT-1; green box in the brain parenchyma and NABT-2; orange box; close to the skull) are shown. In each case a baseline ^2^H MR spectrum (i.e. before oral consumption of [6,6’-^2^H_2_]glucose) and a spectrum obtained ~ 55 min after [6,6’-^2^H_2_]glucose ingestion are shown. The spectra are shown with (white panel) and without (gray panel) PCA-based denoising and zerofilling. The ^2^H MR data is shown in black and the fit is shown in blue

Figure 2 shows examples of ^2^H MR spectra (with and without PCA-denoising) acquired from tumor tissue and in two NABT voxels, one close to the skull and one deeper within the brain parenchyma. In each case, a baseline ^2^H MR spectrum (i.e. before oral consumption of [6,6’-^2^H_2_]glucose) and a spectrum obtained ~ 55 min after [6,6’-^2^H_2_]glucose ingestion is shown. At baseline, a signal from extracranial lipids is evident in the NABT voxel relatively close to the skull. By 55 min post [6,6’-^2^H_2_]glucose ingestion, the signal from ^2^H-Glc is clearly visible in each spectrum. The ^2^H-Glx peak is clearly detectable in NABT, but less apparent in tumor tissue. In contrast, while a small signal from ^2^H-Lac is visible in Tumor tissue, it is not detectable in NABT.

**Fig. 3:**
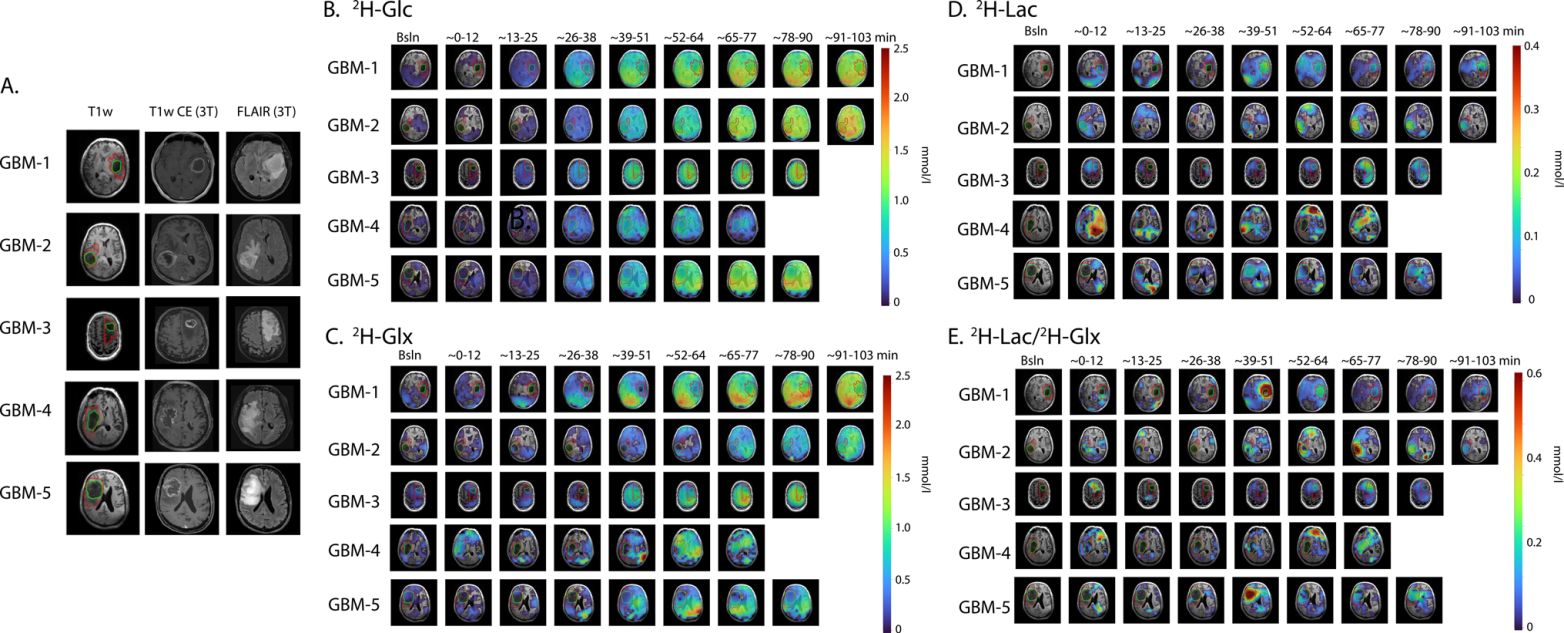
**A** MR images displaying a transversal slice at the location of the tumor. The tumor (red) and tumor core (green) ROI are drawn on the T_1_-weighted image (T_1_w) acquired at 7T. The T_1_-weigthed contrast-enhanced images (T_1_w CE) and the fluid-attenuated inversion recovery (FLAIR) images were acquired at 3T in the weeks prior to the DMI scan. **(B-E)** Dynamic DMI maps in the same transversal slice of ^2^H-Glc **B**, ^2^H-Glx **C**, ^2^H-Lac **D** and ^2^H-Lac/^2^H-Glx **E** after oral consumption of [6,6’-^2^H_2_]glucose

**Fig. 4:**
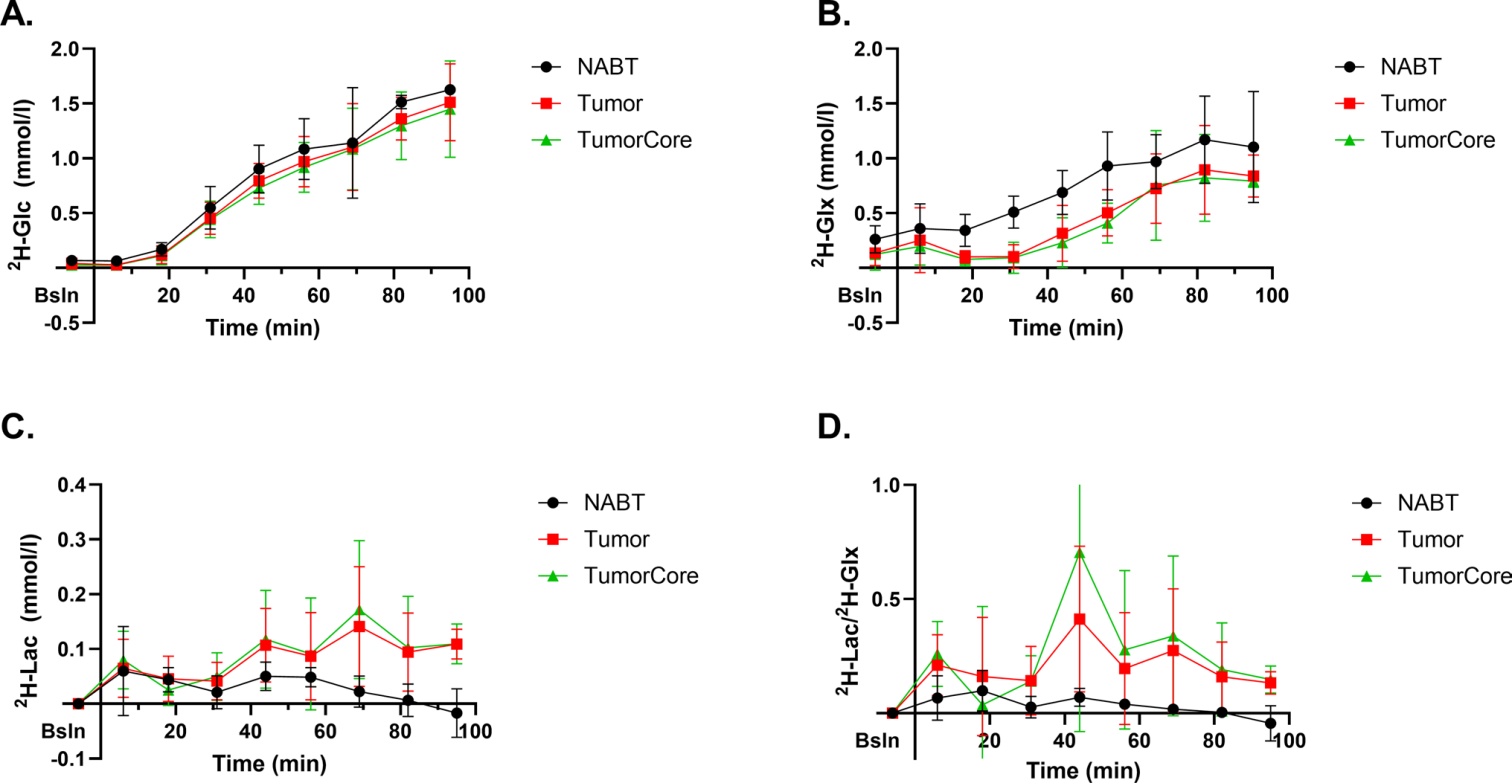
Time curves of mean [± standard deviation] of **A** ^2^H-Glc, **B** ^2^H-Glx, **C** ^2^H-Lac and **D** ^2^H-Lac/^2^H-Glx levels after oral consumption of [6,6’-^2^H_2_]glucose for all patients in normal appearing brain tissue (NABT; black) tumor (red) and tumor-core (green)

Figure 3 displays the dynamic DMI maps, showing levels of brain ^2^H-Glc, ^2^H-Glx, ^2^H-Lac, and the ratio of ^2^H-Lac/^2^H-Glx following the oral consumption of [6,6’-^2^H_2_]glucose. Unprocessed maps (i.e., without spatial smoothing but with PCA-based denoising and zero-filling) are available as supplementary material (Figure S1). Mean ^2^H-Glc, ^2^H-Glx, ^2^H-Lac levels and ^2^H-Lac/^2^H-Glx ratio over time are shown in Fig. 4 for NABT and tumor and tumor-core tissues. Cramer-Rao Lower bounds of the brain ^2^H-Glc, ^2^H-Glx and ^2^H-Lac fits over time are also available as supplementary material (Figure S2).

Brain ^2^H-Glc level increased significantly over time in all patients (*p* < 0.01). There was no statistically significant difference in the increase of brain ^2^H-Glc levels among the three tissue types (*p* = 0.05), nor a significant interaction effect of time x tissue (*p* = 1.0) (Fig. 3, 4). The estimated mean of brain ^2^H-Glc level in all patients by tissue type were (mean ± SE: 0.79 ± 0.03 mM, 0.71 ± 0.03 mM and 0.68 ± 0.03 mM for NABT, tumor and tumor-core, respectively).

Brain ^2^H-Glx level increased significantly over time and was significantly different between the different tissue types (*p* < 0.01). Pairwise comparisons revealed significantly higher ^2^H-Glx levels in NABT compared to both tumor (*p* < 0.01) and tumor-core (*p* < 0.01) tissues, with no significant difference between tumor and tumor-core (p = 1.00) (mean ± SE: 0.70 ± 0.04 mM, 0.43 ± 0.04 mM and 0.39 ± 0.04 mM for NABT, tumor and tumor-core, respectively). Lower ^2^H-Glx levels in tumor tissue compared to NABT were observed consistently across subjects and throughout the entire scan duration. There was no interaction effect of time x tissue (*p* = 0.97).

Brain ^2^H-Lac levels significantly increased over time (*p* < 0.01) and were significantly different between the different tissue types (*p* < 0.01). Pairwise comparison revealed lower ^2^H-Lac in NABT compared to tumor (*p* < 0.01) and tumor-core (*p* < 0.01) (mean ± SE: 0.03 ± 0.01 mM, 0.08 ± 0.01 mM and 0.08 ± 0.01 mM for NABT, tumor and tumor-core, respectively). While ^2^H-Lac levels were elevated in tumor tissue, the signal remained relatively low. In most subjects, detectable levels of ^2^H-Lac in tumor tissue were observed starting at ~ 40 min after consumption of [6,6’-^2^H_2_]glucose. There was no interaction effect of time x tissue (*p* = 0.39). The correction for (extracranial) lipid contamination affects mainly the estimated values in NABT, and only marginally affects the estimated ^2^H-Lac levels in the tumor and tumor-core (Supplementary figure S3).

The ^2^H-Lac/^2^H-Glx ratio exhibited a tumor-specific contrast, starting ~ 40–50 min after oral [6,6’-^2^H_2_]glucose consumption. The duration and persistence of this contrast throughout the scanning period varied between subjects (Fig. 3E, Fig. 4D and S1). There was a significant overall effect of time (*p* < 0.01) and tissue type (*p* < 0.01) for the ^2^H-Lac/^2^H-Glx ratio, but no interaction effect (p = 0.66). The pairwise comparisons indicated significant differences between NABT and tumor (*p* = 0.03) and between NABT and tumor-core (*p* < 0.01), but no significant difference between tumor and tumor-core (*p* = 1.00) (mean ± SE: 0.03 ± 0.41, 0.19 ± 0.041 and 0.23 ± 0.04 for NABT, tumor and tumor-core, respectively).

## Discussion

This study demonstrates the feasibility of dynamic DMI for assessing brain glucose metabolism in GBM patients. We conducted a ~ 100-min dynamic DMI scan, monitoring venous plasma glucose levels, venous plasma ^2^H-Glc APE levels, brain ^2^H-Glc, brain ^2^H-Glx, and brain ^2^H-Lac throughout the scan duration. Our findings show that ^2^H-Glx levels were significantly lower in tumor and tumor-core compared to NABT throughout the time course, suggesting impaired glucose oxidation in tumor tissue. ^2^H-Lac levels were elevated in tumor tissue. The ^2^H-Lac/^2^H-Glx ratio exhibited a tumor-specific contrast, starting at ~ 40–50 min  post [6,6’-^2^H₂]glucose consumption.

A notable finding was the significant difference in ^2^H-Glx levels between NABT and tumor/tumor-core tissues, which lasted for the entire duration of the scan. Tumor and tumor-core exhibited lower ^2^H-Glx levels, suggesting reduced oxidative metabolism. The presence of a ^2^H-Lac peak in tumor tissue, though marginally detectable, indicates active lactate production, consistent with the Warburg effect [3]. This supports the notion that GBM cells favor glycolysis over oxidative phosphorylation, even in the presence of oxygen.

The ^2^H-Lac/^2^H-Glx ratio provided a tumor-specific contrast, aligning with de findings of De Feyter et al. [17]. The contrast was particularly evident starting 40–50 min post-[6,6’-^2^H₂]glucose administration. In these five newly diagnosed GBM patients, the contrast in the ^2^H-Lac/^2^H-Glx map was mainly driven by lower ^2^H-Glx levels in tumor compared to NABT, as ^2^H-Glx levels remained consistently lower in tumor, while ^2^H-lac levels showed greater variability. This contrasts with the study of Feyter et al. in GBM patients who had undergone surgery and chemoradiation [17], which found profoundly increased ^2^H-Lac levels in tumors. In our study, ^2^H-Lac levels in the tumor were lower compared to the study by De Feyter et al., while ^2^H-Glx and ^2^H-Glc levels were within a similar range. These lower ^2^H-Lac levels in tumor cannot be attributed to our lipid correction approach, as this primarily affects ^2^H-Lac/Lipid values in NABT. Interestingly, a recent preclinical study in a glioblastoma mouse model found that a more aggressive GBM subtype exhibited lower ^2^H-Lac accumulation compared to a less aggressive model. This lower ^2^H-Lac accumulation was associated with a higher lactate elimination rate, suggesting increased metabolic plasticity [38]. Furthermore, in that study, ^2^H-Lac accumulation was correlated with lower cell density [38]. In our data, tumor necrosis is clearly visible in the tumor core, which indeed may contribute to lower cell density. Given that we examined newly diagnosed GBM patients, who typically present with more aggressive tumors than post-treatment patients, this metabolic divergence could be attributed to tumor heterogeneity or the presence of necrotic and edematous regions within the tumor ROI.

While a monotonic rise in ^2^H-Lac might be expected, we instead observed a non-monotonic time course with substantial inter-patient variability. We interpret this pattern as the combined result of heterogeneous tumor metabolism, variable lactate clearance, and technical factors such as low SNR, baseline correction effects, and patient motion. In NABT, where ^2^H-Lac levels are very low, occasional small negative values occurred; these are not biologically meaningful and arise possibly because of the correction for baseline ^2^H-lipid/lac levels and subsequent patient movement.

Compared to ^1^H MRSI, which measures static metabolite levels, DMI enables characterization of metabolic fluxes over time, making it possible to assess dynamic downstream glucose metabolism, such as lactate and Glx production. This difference is fundamental, as noted by Brindle (2024), who highlights that only dynamic imaging approaches can reveal ongoing metabolic activity rather than snapshot concentrations [41]. Nonetheless, inclusion of complementary ^1^H MRS measurements would have been informative to determine the total pool sizes of brain metabolites.

As expected, venous plasma glucose levels and venous plasma ^2^H-Glc APE increased in the first ~ 50 min after [6,6’-^2^H₂]glucose ingestion before stabilizing at ~ 60%. The uniform increase in both venous plasma glucose and venous plasma ^2^H-Glc APE across patients indicates a consistent metabolic response to deuterated glucose, and was similar to our previous dynamic DMI study in healthy volunteers [33]. Glucose and ^2^H-Glc APE were measured in peripheral venous blood, which provides a practical estimate of systemic trends. However, since arterial glucose drives cerebral uptake, some deviation from true brain input may occur [39, 40]. Brain ^2^H-Glc levels increased significantly over time, but no significant differences were found between NABT, tumor and tumor-core. This contrasts with ^1^⁸F-FDG-PET studies, which typically show increased glucose uptake in high-grade tumors [42–44]. However, while ^1^⁸F-FDG-PET measures glucose uptake, this does not directly translate to increased glucose accumulation. Our study shows that glucose metabolism is altered downstream, as evidenced by lower ^2^H-Glx labeling and increased lactate production in tumor tissues. DMI thus provides complementary information to ^1^⁸F-FDG-PET, offering insights into glucose metabolism beyond uptake alone.

One of the unique aspects of our data is the dynamic measurement of metabolites in GBM with an extended observation period of ~ 100 min following the oral administration of [6,6’-^2^H₂]glucose. Unlike static imaging, which captures a single time point, dynamic DMI provides a time-resolved view of glucose metabolism, enabling continuous monitoring of metabolic changes over time. Additionally, when aiming for static DMI, identifying the optimal scan timing is crucial; our data suggest that a single scan starting at ~ 40–50 min post-[6,6’-^2^H₂]glucose consumption may be optimal, significantly reducing scan duration while retaining valuable metabolic information. Future studies should explore how metabolic imaging can be integrated into clinical practice, balancing scan duration with diagnostic value.

### Limitations

The small patient cohort poses challenges in interpreting results due to significant variability in individual metabolic profiles and the complexity of GBM metabolism. Furthermore, the DMI voxel size may influence our findings due to the high heterogeneity in GBM tissue and the presence of extensive central necrosis, potentially affecting the results and minimizing the differences between NABT and tumor tissue. This could mask subtle metabolic distinctions, which might be better detected with higher resolution DMI acquisitions, particularly in detecting ^2^H-Lac in tumor tissue. Achieving a smaller voxel within a reasonable scan time might be feasible through acceleration techniques, such as echo-planar spectroscopic imaging (EPSI) [45]. Moreover, ^2^H-Lac levels are very low in NABT, making any statements on dynamics in NABT ambiguous. Another limitation is the need for advanced denoising techniques to improve signal quality, especially for low-level metabolites. Finally, we did not measure the T₁ relaxation times of the ^2^H metabolites in tumor tissue but assumed similar values as have been published previously in healthy volunteers [32]. However, its impact on estimated metabolite levels is expected to be modest. Importantly, ^2^H-Lac levels increased while ^2^H-Glx levels decreased in tumor tissue compared to NABT; opposite trends that are unlikely to result solely from T₁ variations, which would be expected to affect both metabolites in the same direction.

## Conclusion

This study provides insight into the tissue metabolism of GBM prior to any treatment. The tumor-specific contrast in the ^2^H-Lac/^2^H-Glx ratio is mainly driven by lower ^2^H-Glx levels in the tumor compared to NABT. These results highlight the potential of DMI as a non-invasive tool to distinguish metabolic differences between GBM and normal brain tissue.

## Supplementary Information

Below is the link to the electronic supplementary material.

Supplementary file1 (DOCX 1085 KB)

## Data Availability

The data that support the findings of this study are not openly available due to ethical restrictions; participants did not consent to public data sharing, and the data cannot be fully anonymized.
