# Supplementary material for: Dynamic deuterium metabolic imaging in glioblastoma at 7T
Source: MAGMA. 2025 Oct 9;39(3):463–73. doi: 10.1007/s10334-025-01299-3 (PMC13354616; doi:10.1007/s10334-025-01299-3)
Supplement: Supplementary file 1 — Supplementary file1 (DOCX 1085 KB) [file 10334_2025_1299_MOESM1_ESM.docx]

**Supplementary Material**

**
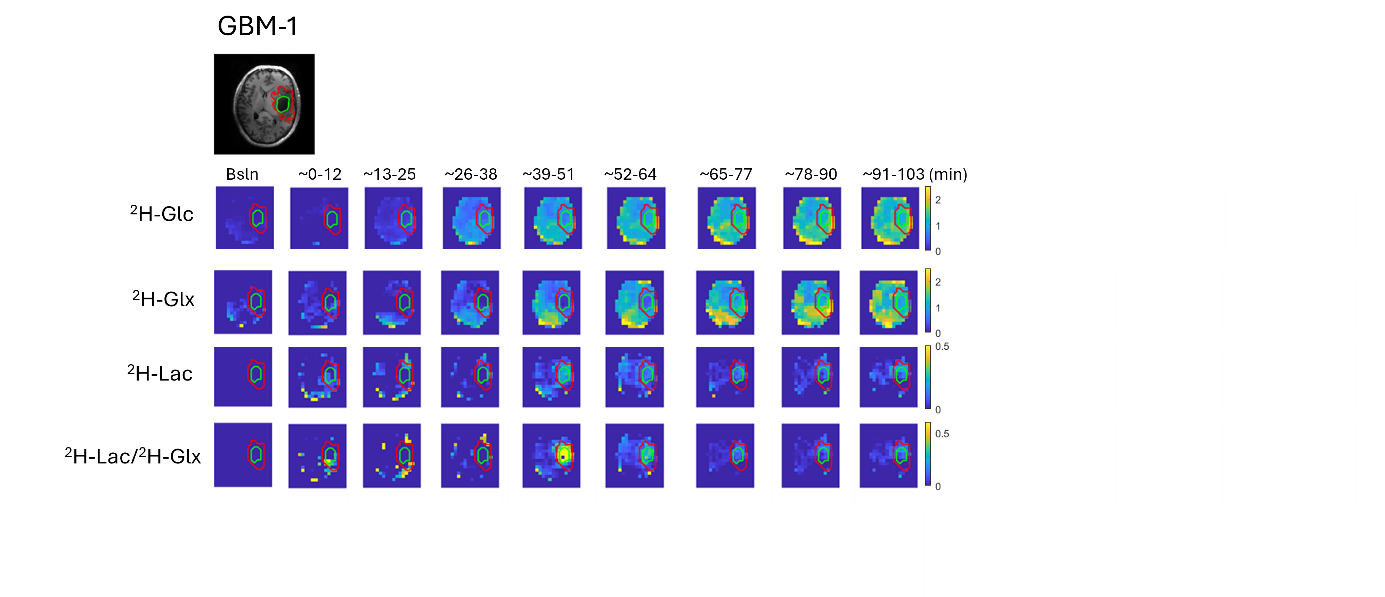

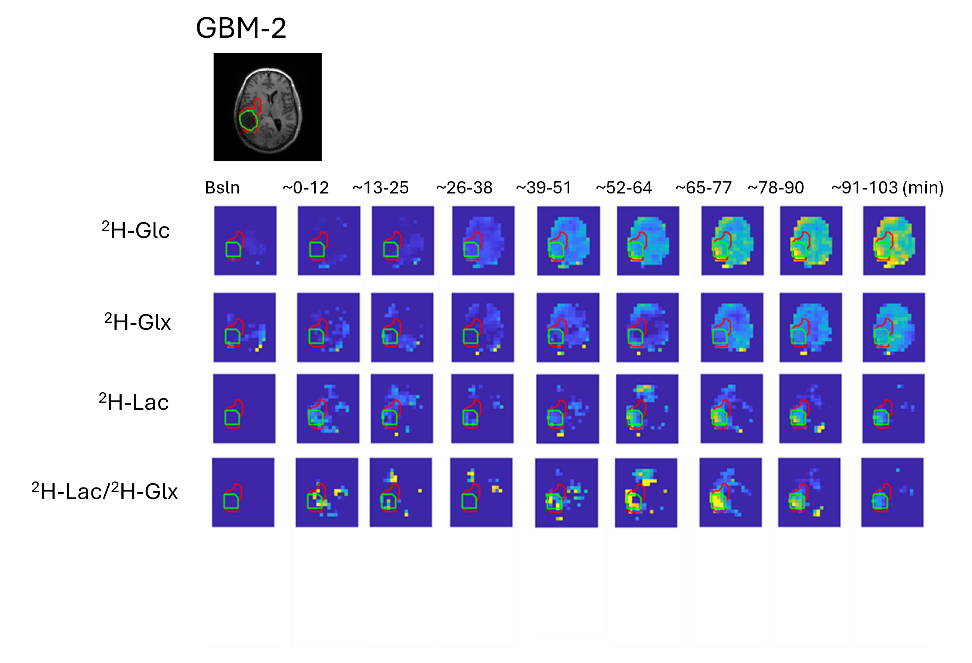

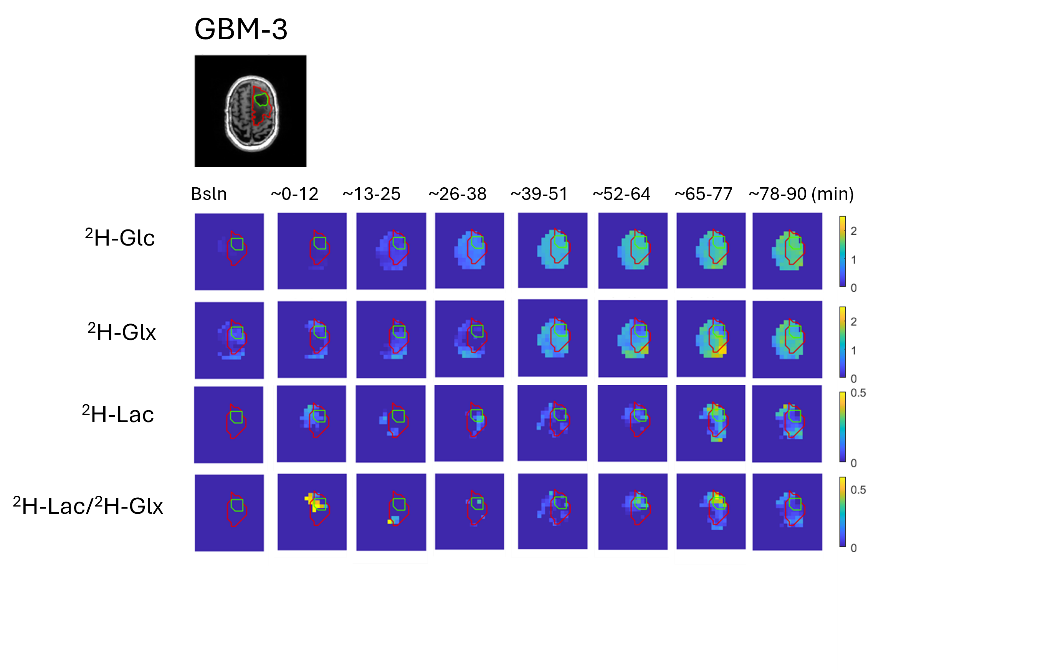

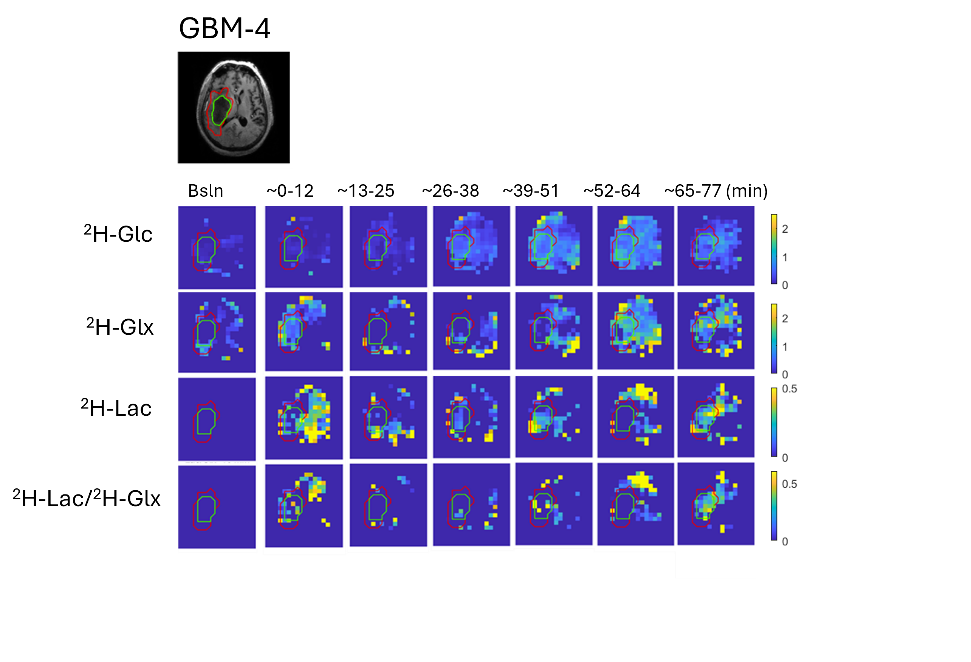
**
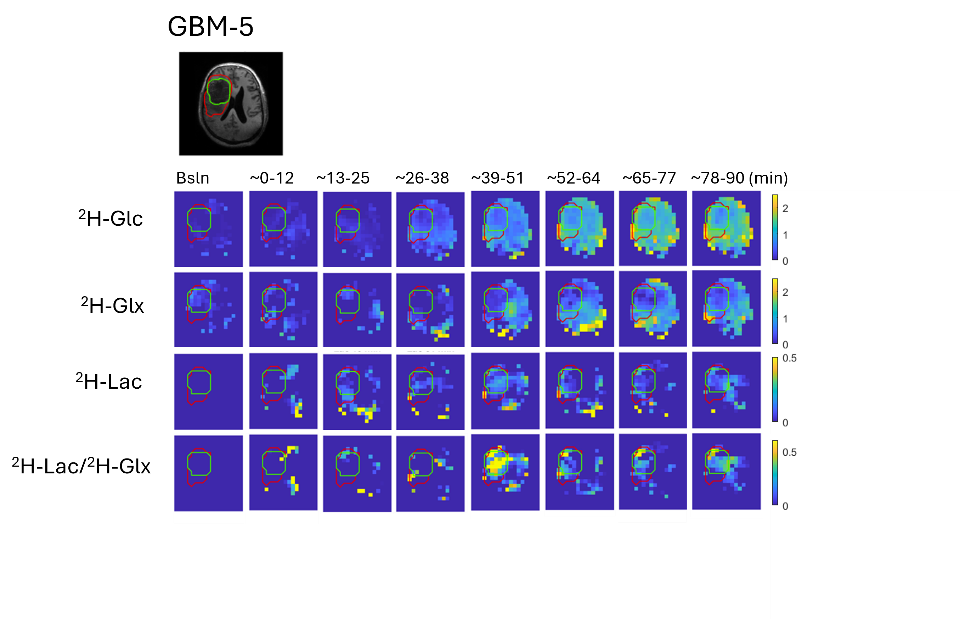


**Figure S1.** Unprocessed **(i.e. non-smoothed)** dynamic DMI maps in the same transversal slice containing the tumor of ^2^H-Glc, ^2^H-Glx ^2^H-Lac and ^2^H-Lac/^2^H-Glx.

**Figure S2.** Cramer-Rao Lower bounds of the metabolite fits (^2^H-Glc, ^2^H-Glx ^2^H-Lac) over time


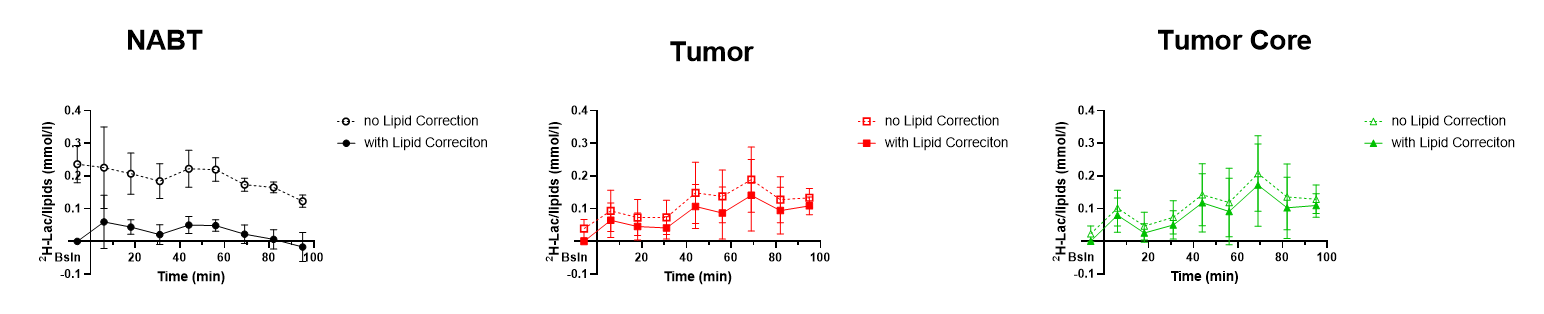


**Figure S-3.** Time course of ^2^H-Lac/Lipid over time in NABT, Tumor and TumorCore with (closed sympols) and without (open symbols) baseline lipid correction
